# Redesigning a clinical mentoring program for improved outcomes in the clinical training of clerks

**DOI:** 10.3402/meo.v20.28327

**Published:** 2015-09-16

**Authors:** Chia-Der Lin, Blossom Yen-Ju Lin, Cheng-Chieh Lin, Cheng-Chun Lee

**Affiliations:** 1 Department of Education, China Medical University Hospital, Taichung, Taiwan, ROC; 2 Department of Otolaryngology, China Medical University Hospital, Taichung, Taiwan, ROC; 3 School of Medicine, China Medical University, Taichung, Taiwan, ROC; 4 Department of Family Medicine, China Medical University Hospital, Taichung, Taiwan, ROC; 5 Department of Healthcare Administration, College of Medical and Health Science, Asia University, Taichung, Taiwan, ROC

**Keywords:** clinical mentoring, clerks, mentoring program, mentor, mentee, medical student, Taiwan, longitudinal survey

## Abstract

**Introduction:**

Mentorship has been noted as critical to medical students adapting to clinical training in the medical workplace. A lack of infrastructure in a mentoring program might deter relationship building between mentors and mentees. This study assessed the effect of a redesigned clinical mentoring program from the perspective of clerks. The objective was to assess the benefits of the redesigned program and identify potential improvements.

**Methods:**

A redesigned clinical mentoring program was launched in a medical center according to previous theoretical and practical studies on clinical training workplaces, including the elements of mentor qualifications, positive and active enhancers for mentor–mentee relationship building, the timing of mentoring performance evaluation, and financial and professional incentives. A four-wave web survey was conducted, comprising one evaluation of the former mentoring program and three evaluations of the redesigned clinical mentoring program. Sixty-four fifth-year medical students in clerkships who responded to the first wave and to at least two of the three following waves were included in the study. A structured and validated questionnaire encompassing 15 items on mentor performance and the personal characteristics of the clerks was used. Mixed linear models were developed for repeated measurements and to adjust for personal characteristics.

**Results:**

The results revealed that the redesigned mentoring program improved the mentors’ performance over time for most evaluated items regarding professional development and personal support provided to the mentees.

**Conclusions:**

Our findings serve as an improved framework for the role of the institution and demonstrate how institutional policies, programs, and structures can shape a clinical mentoring program. We recommend the adoption of mentorship schemes for other cohorts of medical students and for different learning and training stages involved in becoming a physician.

The history of mentoring programs for medical students and doctors can be traced to at least the 1990s (1). Of the various definitions of mentoring, the most widely accepted definition in the scientific literature reviewed by Frei et al. (2) is a process whereby an experienced, highly regarded, empathetic person (the mentor) actively guides another, usually younger person (the mentee), in developing and reexamining his or her own ideas, learning, personal life (e.g., coping with stress and establishing a satisfying work–life balance) (3), and professional development (e.g., career development and research enhancement) (4–6). In addition, mentoring is viewed as a process for informally transmitting knowledge, social capital, and psychosocial support to facilitate communicating the values, vision, and mission of an institution or organization, and thus assisting juniors in understanding the organizational culture and making any necessary changes for workplace socialization (7, 8).

The clerkship is a formal learning stage at a workplace in which students learn a broad range of theoretical knowledge and clinical skills in combination with other competencies. Because clerkships can entail extreme stress (9–12), China Medical University Hospital (CMUH) instituted a clinical mentoring program in 2001 (hereafter referred to as the ‘former clinical mentoring program’) to enhance the learning life of clerks, improve their socialization, and provide counseling services, particularly regarding their clinical learning progress.

The former clinical mentoring program had certain limitations – for example, no regular meeting arrangements existed, mentees hesitated to contact their mentors, mentors were unable to trace their mentees’ performance or provide timely feedback, and insufficient funds were available to mentors for meetings and lunch fees. Moreover, vague mentor role definitions deterred the development of mentoring relationships and contributed to a culture in which the mentoring program was assumed to benefit only the mentees.

Some studies have endeavored to identify potential barriers to improving mentoring program design. For example, studies have revealed barriers involving personal factors, such as insufficient confidence or training to be a mentor; relational difficulties, such as juniors not wishing to appear to require help; and structural and institutional barriers, such as insufficient time allocated for mentoring because of competing personal, administrative, and clinical demands (1, 13–17). More constructively, Keyser et al. (18) proposed a framework for institution and department leaders to document and monitor policies for guiding the mentoring process, including criteria for selecting mentors, incentives for motivating faculty to serve effectively as mentors, recommendations to strengthen the mentor–mentee relationship, and suggested means of improving the professional development of both mentees and mentors.

In the studied setting, we believe that nearly all mentors had an awareness of their roles; however, we lacked a suitable infrastructure by which a mentor could serve as a ‘functional mentor’ from a medical-education or healthcare organizational perspective. In other words, once the mentor–mentee relationship was established, few answers existed regarding what institutional strategies could be followed to minimize the operational barriers to successful mentorships. Therefore, the medical education authority at the CMUH decided to redesign the clinical mentoring program, particularly to assist clerks formally beginning their clinical experiences.

This study assessed the effect of the redesigned clinical mentoring program on mentor performance, from the perspective of the clerks. The objective was to assess the benefits of the redesigned program and identify potential improvements.

## Methods

### Description of the former and redesigned clinical mentoring programs

Healy and Welchert (19) defined mentoring as an activity in which more senior or experienced people who have earned respect and power within their fields take more junior or less experienced colleagues under their care to teach, encourage, and ensure their mentees’ success. This definition is appropriate for the clinical learning stage in the career path of a medical professional; whole-person caring for clerks should consider not only their clinical professional training but also their personal lives, particularly socialization into the profession (20).

In the former clinical mentoring program, the mentors were senior physicians nominated by the chairs of the clinical departments. The mentor was an additional person assigned to the mentees; each mentor was assigned one to three mentees. The mentors met monthly with their mentees and documented their interactions. Expenses incurred by the mentors for the meetings were reimbursed to a maximum of the equivalent of US$70 per month. The mentors were required to submit reports to the CMUH Department of Education to request further assistance for mentees who experienced difficulties in their clinical duties. The mentors were assigned these obligations without receiving any additional compensation.

Some elements of the former clinical mentoring program deserve mention as potential shortfalls. Although the mentors were nominated by their department chair, this did not mean that they were necessarily proficient in mentoring skills; the reasons for nomination could as well have been availability, seniority, or ease of recruitment. Many factors might detract from the benefits of mentoring effectiveness. In addition, because the ratio of mentors to mentees was as low as 1:3, CMUH had to recruit many physicians as mentors, including those with insufficient mentoring skills. Moreover, the limited number of mentees per mentor (three persons) might have reduced peer learning and group sharing. In addition to the mentors having to schedule free time to meet with mentees, the upper-limit reimbursement for mentor–mentee meeting fees (for lunch) might have also implicitly discouraged the mentors and mentees from meeting. Moreover, the absence of compensation for the mentors might have implied that the organization did not recognize their efforts.

On the basis of the literature for effective mentoring program design, and to address the shortfalls in the former clinical mentoring program, mentoring structures were prioritized in the redesigned clinical mentoring program. In addition to the aforementioned mentoring functions, the redesigned clinical mentoring program included considerations regarding mentor qualifications (recruiting criteria) (16), positive and active enhancers for mentor–mentee relationship building (1, 16), timing of mentor performance evaluations, and financial (2, 21) and professional (2, 18, 21, 22) incentives. In the redesigned clinical mentoring program, only physicians recognized as excellent in clinical teaching or in mentoring, as rated by former mentees, and who had taken mentoring skills training at the hospital's Center of Faculty Development, could be mentors. A 1:9 mentor–mentee ratio was mandated to reduce the number of mentors needed and increase the opportunities for peer (mentee) discussion, learning, and sharing. Flexible scheduling between mentors and mentees was encouraged by assistants aiding in making the arrangements (i.e., positive and active enhancers for mentor–mentee relationship building). Mentees were to submit regular and irregular evaluations immediately to their mentors for continuous improvement, instead of the annual evaluation of the former mentoring program (i.e., timing of mentoring performance evaluation). In addition, all of the mentors received a monthly salary equivalent to US$70 per mentee per month for their time and were reimbursed for additional costs associated with mentor–mentee meetings and lunches; this was to encourage more interactions and reduce the mentors’ personal financial burden for their mentees (i.e., financial incentives). Moreover, those who performed well in mentoring earned 2 credit points out of a maximum of 10 for annual teaching evaluations, which were used for future academic promotions (i.e., professional incentives). The CMUH authority is planning on incorporating these credits into an annual bonus (i.e., financial incentives). Table 1 summarizes the major elements used in the redesigned clinical mentoring program and its differences from the former program.

**Table 1:** Major elements employed in the former and redesigned clinical mentoring programs

	Former program		
		
Elements	Characteristic	Vulnerability	Redesigned program
Mentor qualifications: personal characteristics and interpersonal traits (16)	Clinical department chairs had the implicit expectation that all or some senior faculty could serve as capable mentors; and they subjectively assigned physicians to serve as mentors.	Mentoring recruiting criteria were not based on mentor characteristics or traits.	Physicians rated as excelling in clinical teaching or mentoring were qualified to be mentors.
	Ratio of mentors to mentees: 1:3	The low ratio of mentors to mentees led to an increased number of mentors being recruited, increasing the chances of recruiting those who were not well qualified.	Ratio of mentors to mentees: 1:9
Positive and active enhancers for mentor–mentee relationship building (1, 16)	The mentors and mentees were free to schedule their own meetings.	No regulations or incentives for the mentor and mentee to meet because of competing personal, administrative, and clinical demands.	Hospital assistants assisted in making arrangements between mentors and mentees. Time was scheduled for mentor–mentee meetings.
Timing of mentor performance evaluation	Annual evaluation	Poor mentor performance may not be assessed in time.	Mentees submitted regular and irregular evaluations immediately to their mentors to facilitate continual improvement.
Financial incentives (2, 21)	Mentors were reimbursed the cost of mentor–mentee meetings up to the equivalent of US$70 per month. Mentors absorbed any financial burden beyond the monthly maximum.	The upper-limit reimbursement for mentor–mentee meeting fees (for lunch) implicitly discouraged meetings. The absence of compensation for mentors implied that the organization did not recognize mentoring efforts.	All mentors received a monthly salary of the equivalent of US$70 per mentee per month. Reimbursements were available for additional costs associated with mentor–mentee meetings.
Professional incentives (2, 18, 21, 22)	None	No professional recognition was identified.	Mentoring outcomes were included in the mentor's annual performance appraisal. Mentors who performed well received extra credit toward future academic promotions.

### Setting and participants

CMUH is a general hospital with 2,047 beds and 3,722 staff (including 374 visiting physicians and 285 residents). CMUH, an affiliated hospital of China Medical University (CMU), provides various primary, secondary, and tertiary care services in Taiwan. Over the past 5 years, the average monthly volume of outpatients and inpatients was 138,498 and 5,554, respectively. All CMU medical students must complete their clerkship at the CMUH in the fifth year of their 7-year medical degree program. A quantitative survey, including one pre-evaluation and three post-evaluations of the redesigned clinical mentoring program, was conducted among all CMU fifth-year medical students in clerkships from September 2013 to May 2014.

### Study population and data collection

One hundred and eighteen medical students were enrolled in the study and provided informed consent. Each clerk was assigned to a mentor at the beginning of his or her clerkship in September 2013, at which time the former mentoring program was still in effect. The first wave of the survey was conducted in early November (T1) to obtain the clerks’ perceptions of the mentoring program without informing them of the upcoming redesigned program. After T1, in mid-November 2013, the redesigned clinical mentoring program was launched. The subsequent three waves of the survey were conducted to evaluate the redesigned mentoring program in December 2013 (T2, 1 month after the redesigned program launch), February 2014 (T3, 3 months after the launch), and May 2014 (T4, 6 months after the launch). Clerks who responded to the first wave and to at least two of the three following waves were included in the study. In total, the sample comprised 64 clerks and 229 responses. The participants did not differ significantly in age or sex from the clerks not included in the study. Ethical approval was granted by the CMU and CMUH Ethics Committees. All data were collected and analyzed after the cohort was established.

### Survey instruments

This study assessed the effect of the redesigned clinical mentoring program from the perspective of the clerks. The objective was to assess the benefits of the redesigned program and identify potential improvements. The respondents were led by an adjunct section to constrain their opinion of their clinical mentor in the clerkship, who was different from the mentor that they had in school. The 15-item mentorship scale of Scandura and Ragins, which encompasses mentoring functions such as psychosocial support, career development, and role modeling (23) was adapted by adding the phrase ‘in your clerkship’ before each question item. Responses were provided on a 5-point Likert scale, with 1 as ‘strongly disagree’, 2 as ‘disagree’, 3 as ‘fair’, 4 as ‘agree’, and 5 as ‘strongly agree’. The scale instrument covered the functions of mentoring from a whole-person perspective that were integrated into the redesigned program (6, 7, 24, 25). A factor analysis revealed 2 major dimensions among these 15 items, namely ‘professional development’ and ‘personal support’. The factor loadings ranged from 0.69 to 0.91 for professional development with a Cronbach's *α* value of 0.97, and from 0.81 to 0.85 for personal support with a Cronbach's *α* value of 0.85. In addition, the personal characteristics of the clerks, including sex and age were measured. The same items were included in each of the four waves of surveys.

### Statistical analysis

Descriptive analyses were used to establish the baseline characteristics of the participants. Because the pre- and post-evaluations of the former and redesigned mentoring programs of each participant as cohorts were not independent (i.e., they were correlated data), did not exhibit unequal variance, and involved an unequal number of repetitions, a linear, mixed-effects model was used (26, 27). With individual mentoring items as dependent variables, the four evaluations of the mentoring programs (i.e., T1, former mentoring program evaluation and T2–T4 redesigned mentoring program evaluations) were independent variables, with T1 as the baseline reference and the age and sex of the clerks as covariates. All analyses were performed using SPSS Version 20.0 and Excel was used to plot the data.

## Results

In total 64 clerks, 37 men (58%) and 27 women (42%) with an average age of 23.63 years, completed one evaluation of the former mentoring program (T1) and at least two evaluations of the redesigned clinical mentoring program (T2–T4) during the study period. The T1, T2, T3, and T4 evaluations were completed by 64, 53, 56, and 56 participants, respectively.

**Fig. 1:**
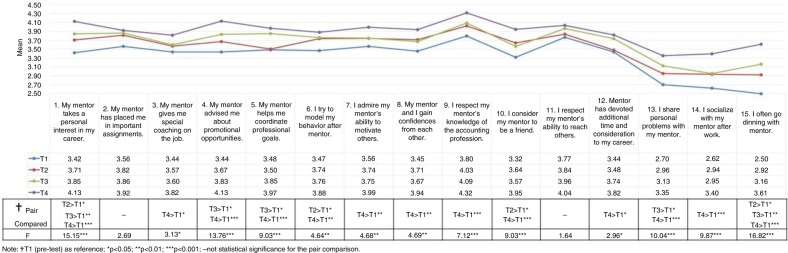
Four-wave mentoring evaluations.

In general, from the T1 baseline, evaluations of 13 out of the 15 items improved progressively from T2 to T4, particularly at the final evaluation (T4) (*p<*0.05) (Fig. 1). Regarding professional development (Items 1–12), the mentees reported a steady improvement in mentor guidance in personal career interests (Item 1), promotional opportunities (Item 4), and coordinating professional goals (Item 5) (*p*<0.05). The evaluation items regarding the mentors’ special, on-the-job coaching (Item 3), the mentors’ ability to motivate others (Item 7), the mentors’ devotion of additional time and consideration of the mentees’ careers (Item 12), the mentees’ confidence in their mentors (Item 8), and the mentees’ respect of their mentors’ knowledge of the accounting profession (Item 9) did not increase initially after the redesigned clinical mentoring program (T2 and T3, *p*>0.05), but did 6 months after the program was implemented (T4, *p*<0.05). The mentees’ modeling of their behavior after the mentors (Item 6) and viewing their mentors as friends (Item 10) did not receive progressively improved evaluations over time (*p*>0.05); however, they were finally rated the highest 6 months after the redesigned program was implemented (T4, *p*<0.05).

Regarding personal support (Items 13–15), the mentees rated sharing personal problems (Item 13) and dining after work (Item 15) progressively higher over time (*p*<0.05). In addition, the mentees reported socializing more with their mentors (Item 14) 6 months after the program was implemented (T4) (*p*<0.05).

After the implementation of the redesigned program, two items with no improvement were observed. One involved the mentees’ evaluation of how the mentors placed them on critical assignments (Item 2) and the other involved the mentors’ ability to teach others (Item 11); these two items exhibited no statistically significant differences over time (T1–T4, *p*>0.05). In addition, we observed no statistically significant differences in sex or age regarding the mentees’ evaluation of their mentors’ performance (*p*>0.05).

## Discussion

For the past several decades, job design and redesign have been perceived in organizational behavior as factors that influence the psychological job statuses and outcomes of employees. When designing and redesigning a job, such as the mentoring of clinical physicians, attempts are frequently made to identify the critical needs of mentors and remove potential obstacles to those needs, all of which contribute to the effectiveness of individual mentors and medical education as a whole. In this study, we conducted only one evaluation of the former mentoring program (T1). It is questionable whether there would have been an improvement over time regardless of the new program. Although only one wave of evaluation for the former program was conducted, some shortfalls had already been identified by previous mentors and mentees, which was why the medical education authority of CMUH decided to redesign the mentoring program. The clinical mentoring program that we redesigned was based on previous studies that included the elements of mentor qualifications, positive and active enhancers for mentor–mentee relationship building, the timing of mentoring performance evaluation, and financial and professional incentives, which had assumed benefits according to theoretical perspectives in organizational behavior.

We determined that, overall, the redesigned program improved mentor performance over time for most of the evaluated items, as rated by the mentees. Although the personal support scores increased significantly (Items 13–15, Fig. 1), they were still lower than the professional development scores (Items 1–12, Fig. 1). Keyser et al. (18) argued that compatibility between the mentor and mentee is critical to a successful mentoring relationship; however, no definitive conclusions exist regarding whether assignment is superior to voluntary matching in mentor–mentee relationships. Cappell (28) argued that a mentor–mentee relationship should be formed by mutual consent. In our redesigned clinical mentoring program, we did not permit mentees to freely select their mentors, but mentor qualifications were enhanced when mentors were selected according to criteria such as being ranked as a physician of excellence in clinical teaching or mentoring. We speculate that an assigned mentor–mentee relationship requires time for mutual trust to develop; thus, more time would be required for establishing a deeper psychosocial relationship in which the mentee perceives the mentor as offering personal support. This possibility leaves room for improving our program design. In addition, we found that evaluations of the item ‘My mentor has placed me on important assignments’ (Item 2) did not increase after the redesigned clinical mentoring program was launched. We attribute this result to the mentors’ many tasks, such as clinical services, teaching, and research, in addition to their mentoring tasks, which might have given the mentees the impression that the mentoring tasks were relatively less critical assignments for the mentors. Moreover, the item ‘I respect my mentor's ability to teach others’ (Item 11) did not receive increased ratings over time. This result might be attributable to several factors, including the mentees having no opportunities to observe their mentors teaching others and the mentoring functions in the redesigned clinical mentoring program not being focused on teaching tasks but on a whole-person perspective for the mentees. We will continue to identify other possible reasons.

Some limitations deserve mention. We did not make any direct one-cause-to-one-effect evaluations; rather, we assessed the entire package of redesigned elements as a whole. In addition, the survey instrument used in this study was focused on mentor performance. Future empirical studies can explore other objectives of the clinical mentoring program such as the mentor–mentee relationship in the short and long terms; this relationship might influence clerks’ careers. This study also did not weigh the individual effects of different factors in the redesigned mentoring program, such as whether financial or professional incentives were the key factors behind improved mentor performance. The implications of financial incentives in mentoring programs should be addressed in future studies. Some role models who meet clerks in other settings, such as in extracurricular activities or medical events outside of the workplace, might be viewed as informal mentors and these influences should also be explored. We will promote and publicize this redesigned clinical mentoring model for other stages of medical student training, such as internships and postgraduate physician residencies. Future studies can examine mentor perceptions of the redesigned clinical mentoring program to improve its effectiveness from mentor and job design perspectives.
